# Development and validation of a nomogram prediction model for perioperative delirium in older patients with osteoporotic fractures based on LASSO regression and multiple parameters

**DOI:** 10.3389/fmed.2026.1869420

**Published:** 2026-09-03

**Authors:** Ni Hua, Hongmei Yuan, Yanhong Wei, Zhenqi Wei, Yanping Feng, Yong Liu, Tongguang Xu, Suliang Zhao, Li Zhang, Xiaohong He

**Affiliations:** 1 Department of Orthopedics, The People’s Hospital of SND, Suzhou, Jiangsu, China; 2 The People’s Hospital of SND, Suzhou, Jiangsu, China; 3 Hand and Foot Joint Surgery, The People’s Hospital of SND, Suzhou, Jiangsu, China; 4 Spine Surgery, The People’s Hospital of SND, Suzhou, Jiangsu, China; 5 Department of Nursing, The People’s Hospital of SND, Suzhou, Jiangsu, China

**Keywords:** delirium, machine learning, nomogram, osteoporotic fracture, risk factors

## Abstract

**Background:**

Perioperative delirium (POD) is a severe postoperative complication in older patients with osteoporotic fractures. This study aimed to establish a nomogram prediction model incorporating multisystem stress, inflammation and frailty-related biomarkers for risk prediction.

**Methods:**

This retrospective cohort study included 176 older patients (≥65 years) with osteoporotic fractures. Potential predictors, including the rate-pressure product (RPP), systemic inflammation response index (SIRI) and prognostic nutritional index (PNI), were analysed. Least absolute shrinkage and selection operator regression was utilised for feature selection to construct a multivariable logistic regression nomogram model, which was internally validated via bootstrap resampling.

**Results:**

Least absolute shrinkage and selection operator regression and logistic regression identified six independent predictors of POD: age, preoperative dementia (cognitive dysfunction), elevated admission RPP, elevated SIRI, decreased PNI and prolonged postoperative intensive care unit (ICU) stay. These indicators serve as predictive markers rather than confirmed causal factors for POD. The nomogram model presented favourable discriminative ability (area under the curve >0.85). The calibration curve showed favourable consistency between the predicted probability of POD and the actual incidence. Decision curve analysis indicated that the model might yield a potential net clinical benefit.

**Conclusion:**

A prediction model integrating RPP, SIRI and PNI showed favourable performance in assessing POD risk among older patients with osteoporotic fractures, which may provide a reference for clinicians to conduct individualised pre-emptive interventions. However, interpretation of model performance should be conducted with caution given the inherent limitations of this study.

## 1 Introduction

Under the macroscopic background of global population ageing, osteoporosis and its concurrent fragility fractures have become a major public health challenge facing the global healthcare system. Epidemiological data predict that the global incidence of osteoporotic hip fractures will double by 2050, with Asia being particularly severely affected by this disease (1, 2). For older patients with osteoporotic fractures (especially hip fractures and vertebral compression fractures), surgery is currently the preferred treatment for relieving pain and restoring anatomical structure and function. However, older patients generally present with complex conditions such as reduced physiological reserve, multiple underlying comorbidities (multimorbidity) and polypharmacy, making them sensitive to perioperative stress. Among numerous postoperative complications, perioperative delirium (POD), due to its high incidence and severe clinical harm, has become an urgent problem to be solved in the fields of geriatric orthopaedics and anaesthesiology (3, 4).

Delirium is a neuropsychiatric syndrome characterised by attention deficits, altered levels of consciousness and acute fluctuations in cognitive function. In the older population, the rate of missed diagnosis is high due to the insidious onset of some delirium symptoms (5). The literature reports that the occurrence of postoperative delirium in older patients with hip fractures not only considerably prolongs the hospital stay and sharply increases medical and nursing costs but also substantially elevates the risk of complications such as postoperative infections. More seriously, POD is an independent risk factor for accelerating long-term cognitive decline, inducing dementia and substantially increasing all-cause mortality in older patients (6).

Although the clinical harm of POD is profound, its exact pathogenesis has not yet been fully elucidated. The currently widely accepted theory in the academic community is the “predisposing–precipitating” mechanism model, in which intense perioperative stress events (such as trauma, pain and anaesthetic drugs) are superimposed on the patient’s existing vulnerabilities (such as advanced age, brain atrophy, preoperative cognitive dysfunction and malnutrition), ultimately triggering an acute breakdown of the central nervous system network. In this process, the neuroendocrine stress hypothesis and the neuroinflammation hypothesis occupy a core position (7). Severe pain and physiological stress caused by fracture trauma and surgical procedures may induce sympathetic nervous system activation and a massive release of catecholamines, further disrupting cerebral blood flow microcirculation and cerebral metabolism. The rate-pressure product (RPP) is a clinical indicator used to quantify perioperative physiological stress and is associated with elevated POD risk (8). Simultaneously, severe peripheral tissue injury may trigger a systemic inflammatory response. The systemic inflammation response index (SIRI) is correlated with POD occurrence in this patient population (9).

Since there are currently no specific therapeutic drugs for POD, the accurate identification of high-risk populations and the implementation of preventive interventions have always been the optimal strategy. In recent years, although several studies on POD prediction models have been published (10–12), existing models still have major limitations. Most models focus only on single-dimensional indicators, ignoring the multi-system cascade reactions of the human body as a complex system under stress conditions (13), and they are highly prone to falling into the trap of multicollinearity and model overfitting when processing high-dimensional clinical data, leading to a sharp decline in the external validation efficacy of the models.

To overcome existing research shortcomings, least absolute shrinkage and selection operator (LASSO) regression (14–16) was applied to perform dimensionality reduction and feature selection from high-dimensional perioperative data. Markers related to stress, inflammation and physical reserve (RPP, SIRI, neutrophil-to-lymphocyte ratio [NLR], prognostic nutritional index [PNI]) (8, 17–20) and standard baseline data were incorporated. A practical nomogram for POD risk estimation in older patients with osteoporotic fractures was finally established and validated. This study focuses on clinical risk prediction rather than exploring POD’s causal pathogenesis. The perioperative prediction model is not limited to preoperative assessment but incorporates postoperative variables to better predict POD within 7 days after surgery.

## 2 Materials and methods

### 2.1 Study design and participants

This was a retrospective observational cohort study, with data sourced from the electronic medical record (EMR) system of the study centre for older patients with osteoporotic fractures admitted in recent years. The implementation and reporting of this study strictly followed the Transparent Reporting of a multivariable prediction model for Individual Prognosis or Diagnosis statement and its artificial intelligence/machine learning extension guidelines (21, 22), ensuring scientific rigour in variable selection, model construction and performance evaluation. This study complied with the principles of the Declaration of Helsinki and was approved by the medical ethics committee of the study hospital (Approval No.: 2026-038). All patients and their families were informed of the study purpose and potential risks before surgery and signed informed consent forms.

The inclusion criteria were as follows: (1) aged ≥65 years; (2) received a definitive clinical and imaging diagnosis of an osteoporotic fracture and undergoing elective or emergency surgery; and (3) had complete perioperative clinical data, baseline vital signs and laboratory test results.

The exclusion criteria were as follows: (1) patients with a history of severe schizophrenia or who were in a state of severe aphasia or coma and were unable to cooperate with the delirium assessment scale test, (2) patients with pre-existing end-stage multiple organ failure and an expected life expectancy of less than 3 months before surgery and (3) cases with missing core clinical data exceeding 20%.

Based on the above criteria, a final cohort of 176 eligible patients was included in the statistical analysis.

### 2.2 Data collection and multidimensional parameter definition

Potential predictor variables covering the patients’ multisystem status were systematically extracted by independent researchers, including:

1) Baseline demographic and clinical characteristics: Age, sex, body mass index (BMI), smoking and drinking history, preoperative comorbidities (hypertension, diabetes, cardiovascular and cerebrovascular diseases, etc.) and the presence of preoperative mild cognitive impairment or a history of dementia.
2) Preoperative physiological stress indicators: The first resting heart rate and systolic blood pressure recorded on admission were extracted to calculate the RPP. The calculation formula was as follows: RPP = admission resting heart rate × admission systolic blood pressure.
3) Systemic immune-inflammatory and nutritional indicators: Preoperative routine blood and biochemical test results were extracted to calculate the following derived indices:

$$\begin{array}{l} \text{Systemic inflammation response index}\\ =(\text{neutrophil count}\times \text{monocyte count})/\text{lymphocyte count} \end{array}$$

$$\begin{array}{l} \text{Neutrophil}-\text{to}-\text{lymphocyte ratio}\\ =\text{neutrophil count}/\text{lymphocyte count} \end{array}$$

$$\begin{array}{l} \text{Prognostic nutritional index}=\text{serum albumin}\;(g/L)\\ +5\times \text{total peripheral blood lymphocyte count}\;(10^{9}/L) \end{array}$$

4) Perioperative and surgery-related parameters: American Society of Anesthesiologists physical status classification, surgical delay, anaesthesia method, operation duration, estimated intraoperative blood loss and postoperative ICU stay duration.

### 2.3 Diagnostic criteria for delirium and outcome assessment

The primary outcome of this study was the occurrence of POD within 7 days postoperatively. Delirium assessments were independently performed by two trained geriatricians certified in the Confusion Assessment Method (CAM) and three-dimensional CAM (3D-CAM), both blinded to the patients’ laboratory results and RPP, SIRI and PNI values (5). According to the diagnostic criteria, a patient was diagnosed with POD if they concurrently presented with (1) acute onset and fluctuating course and (2) inattention, together with either (3) disorganised thinking or (4) an altered level of consciousness. Standardised training included a 4-h workshop with 20 video-based case scenarios (*κ* = 0.91 for inter-rater reliability). Assessments were conducted twice daily (08:00 and 20:00) during the first 7 postoperative days. Borderline or fluctuating cases were jointly reviewed by both assessors, with a consensus diagnosis required. Hypoactive delirium was systematically captured using the full 3D-CAM algorithm rather than isolated observation. Inter-rater reliability was reassessed every 3 months throughout the study (mean *κ* = 0.89, 95% confidence interval [CI]: 0.84–0.93).

### 2.4 Statistical analysis and prediction model construction

All data cleaning and statistical analyses were performed using R software (version 4.2). Variables with missing data <10% included serum albumin (3.2%), lymphocyte count (2.8%), monocyte count (2.8%), admission heart rate (1.7%) and admission systolic blood pressure (1.1%). Missing data were imputed using the multivariate imputation by chained equations algorithm with 20 imputations, incorporating age, sex, BMI, fracture type, preoperative cognitive status and admission laboratory values as predictors in the imputation model. Variables with missingness ≥20% (e.g., intraoperative bispectral index [BIS] values, 24.6%) were excluded before modelling. To assess robustness, a complete-case sensitivity analysis was performed (*n* = 168). Model coefficients and the area under the curve (AUC) were compared between the imputed and complete-case datasets, with all changes <5% and no statistically significant differences (ΔAUC = 0.012, *p* = 0.680).

For feature selection, LASSO regression was performed using a total of 24 candidate variables initially included in the analysis. Given the limited sample size and the relatively small number of POD events, LASSO regression with L1 regularisation was specifically adopted to constrain model complexity, shrink redundant variable coefficients to zero and effectively reduce the risk of overfitting caused by a high ratio of candidate variables to outcome events. Ten-fold cross-validation was applied to determine the optimal penalty parameter, which further ensured the parsimony and generalisability of the final model. Notably, postoperative intensive care unit (ICU) stay duration was retained in the final model after LASSO regression feature selection. Although this indicator is measured after surgery, it is related to the overall perioperative physiological status and environmental stress of patients. Given that the primary endpoint, POD, is defined as events occurring within 7 days postoperatively, the inclusion of this perioperative indicator helps enhance the predictive performance for late-onset POD.

Nomogram construction and model evaluation: The predictors selected by LASSO regression were incorporated into a multivariable logistic regression model to construct a nomogram predicting the individual probability of POD. The validation of model performance focused on the following dimensions:

1) Discrimination: Receiver operating characteristic curves were plotted, and the AUC (or C-index) was calculated to assess the model’s discriminative ability.
2) Calibration: Given the sample size limitations, the bootstrap resampling method (1,000 resamples) was used for rigorous internal validation and to plot calibration curves. The goodness-of-fit was evaluated using the Hosmer–Lemeshow test.
3) Clinical utility: Decision curve analysis (DCA) was used to evaluate the net clinical benefit of the nomogram model at different threshold probabilities.

## 3 Results

### 3.1 Baseline characteristics of the cohort

A total of 176 older patients undergoing surgery for osteoporotic fractures were included in this study. Among them, 22 patients (12.5%) developed POD, and 154 patients (87.5%) did not develop it. The average age of the POD group was significantly higher than that of the non-POD group. Regarding comorbidities and baseline status, the proportion of patients with preoperative cognitive dysfunction or a history of dementia in the POD group was significantly higher than that in the non-POD group. Furthermore, in terms of admission physiology and laboratory tests, patients in the POD group exhibited more intense stress responses and inflammatory states than patients in the non-POD group, specifically manifested as significantly elevated RPP, SIRI and NLR at admission. Reflecting nutritional status, PNI was significantly lower in the POD group than in the non-POD group. Among the perioperative variables, the POD group had greater intraoperative blood loss and a longer ICU stay. The specific comparison of baseline characteristics between the two groups is shown in Table 1. Among the 176 patients, 98 (55.7%) received general anaesthesia, 49 (27.8%) received regional anaesthesia and 29 (16.5%) received combined anaesthesia. Intraoperative anaesthetic depth was routinely monitored using the BIS, maintaining a target range of 40–60 throughout surgery; BIS data with missingness exceeding 20% were excluded from the primary regression modelling. No postoperative sedative agents were administered to any patients intraoperatively or after surgery. All participants received standardised postoperative analgesia centred on twice-daily intravenous ketorolac tromethamine infusion; only a small subset of patients additionally received intravenous patient-controlled analgesia pumps for rescue pain relief. Intraoperative benzodiazepines were administered in 31 (17.6%) patients, intraoperative opioids in 162 (92.0%) and anticholinergics in 44 (25.0%). Intraoperative hypotension occurred in 38 (21.6%) patients, hypoxaemia in 12 (6.8%) and allogeneic blood transfusion in 27 (15.3%). Postoperative infections developed in 14 (8.0%) patients, and non-delirium systemic complications occurred in 21 (11.9%). Fracture sites comprised 112 (63.6%) hip fractures, 49 (27.8%) vertebral compression fractures and 15 (8.5%) other osteoporotic fractures. Preoperative functional status was assessed using the Barthel Index (median 65 [interquartile range (IQR): 50–80]), and frailty was assessed using the Fried Frailty Phenotype (52 [29.5%] prefrail, 37 [21.0%] frail). Baseline cognitive function was evaluated using the Mini-Mental State Examination, with a median score of 24 (IQR: 20–27); 28 (15.9%) patients had a prior diagnosis of dementia. The median time from fracture to surgery was 3 days (IQR: 2–5). All patients received standardised multimodal analgesia (acetaminophen plus opioid patient-controlled analgesia), and no centre-wide prophylactic delirium protocols were implemented during the study period.

**Table 1:** Comparison of baseline data between the POD group and non-POD group in elderly patients with osteoporotic fractures.

Characteristic variables	All patients (*N* = 176)	POD group (*N* = 22)	Non-POD group (*N* = 154)	t/χ2/Z	*p*-value
Age (years, mean ± SD)	72.04 ± 6.49	84.27 ± 7.81	70.29 ± 3.90	8.25	<0.001
Sex (Male/Female, *n*)	50/126	4/18	46/108	0.78	0.376
Preoperative dementia/cognitive impairment [*n* (%)]	4 (2.3%)	4 (18.2%)	0 (0.0%)	21.05	<0.001
Admission RPP (mmHg·bpm)	10050.93 ± 1942.39	11207.86 ± 2826.76	9885.65 ± 1731.68	2.14	0.043
SIRI (M [IQR])*	1.31 [0.71–2.41]	3.23 [2.20–4.89]	1.15 [0.67–2.05]	2,803	<0.001
NLR (M [IQR])*	2.99 [1.92–4.40]	6.54 [4.14–11.62]	2.70 [1.87–4.09]	2,894	<0.001
PNI (mean ± SD)	49.99 ± 6.50	43.40 ± 3.27	50.94 ± 6.30	−8.75	<0.001
Operation duration (min)	91.62 ± 53.67	86.82 ± 61.62	92.31 ± 52.62	−0.4	0.694
Intraoperative blood loss (ml)	123.61 ± 161.29	354.55 ± 272.08	90.62 ± 104.07	4.5	<0.001
Postoperative ICU stay (h)*	0.00 [0.00–0.00]	0.09 [0.00–0.00]	0.00 [0.00–0.00]	1,848	<0.001

*SIRI, NLR, and ICU stay are described using the median [interquartile range] due to their skewed distribution.

### 3.2 Least absolute shrinkage and selection operator regression for predictive feature selection

**Figure 1:**
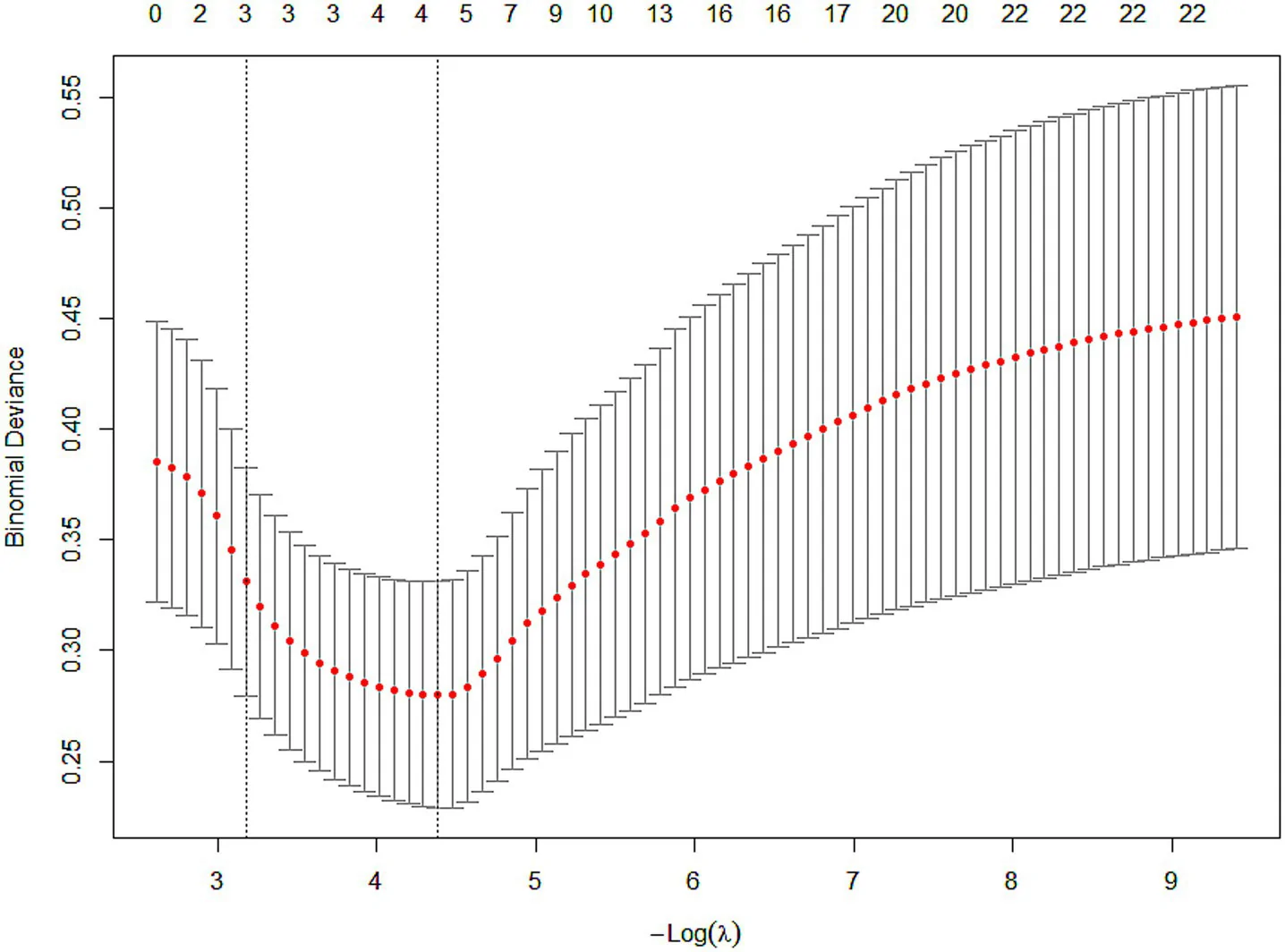
LASSO cross-validation curve for feature selection. The trajectory of partial likelihood deviance changing with the penalty parameter log(*λ*) was plotted via 10-fold cross-validation for LASSO regression. The two dashed lines indicate the *λ* value corresponding to the minimum prediction error and the *λ* value at one standard error of the minimum error (*λ* 1se). The *λ* 1se criterion was adopted to screen variables and obtain a parsimonious feature subset for subsequent modelling.

In this study, LASSO regression was used to perform dimensionality reduction on 24 candidate clinical characteristics and laboratory parameters. As the penalty parameter increased, the coefficients of redundant variables shrank to zero. The LASSO model successfully eliminated redundant and collinear variables, ultimately retaining several variables with the strongest predictive value and non-zero coefficients (see Figure 1). These variables were confirmed as key factors for predicting POD: age, preoperative cognitive dysfunction (history of dementia), admission RPP, SIRI, PNI and postoperative ICU stay. The final events-per-variable ratio was 3.67 (22 POD events/6 predictors). Although this ratio was suboptimal according to conventional statistical criteria, the combination of LASSO penalisation and multiple cross-validation effectively mitigated the corresponding risk of overfitting. The optimal penalisation value (*λ*) determined by 10-fold cross-validation was 0.032. All variables and the corresponding screening details are listed in Supplementary Table S1.

### 3.3 Multivariate logistic regression and establishment of independent risk factors

The six feature variables selected by LASSO regression were incorporated into a multivariable logistic regression analysis. The analysis confirmed that all six variables were independent risk factors for POD in older patients with OPF (all *p* < 0.05). Among them, pre-existing dementia or preoperative cognitive dysfunction was a predictor, reflecting the patient’s vulnerable neural reserve. Each unit increase in admission RPP was independently associated with a higher risk of POD. Elevated SIRI was also an independent predictive factor for increased POD risk, whereas a higher PNI was independently associated with a lower risk of POD. All these indicators are effective predictive markers of POD. The complete results of the multivariable logistic regression model, including odds ratios, 95% CIs and beta coefficients for each independent predictor, are summarised in Table 2.

**Table 2:** Results of multivariate logistic regression for predictors of POD.

Variable	Beta coefficient	Odds ratio (OR)	95% CI for OR	*P*-value
Age	0.086	1.090	1.042–1.139	<0.001
Preoperative cognitive dysfunction/dementia	1.624	5.073	2.116–12.152	<0.001
Admission RPP	0.0002	1.0002	1.0000–1.0004	0.043
SIRI	0.415	1.514	1.207–1.898	<0.001
PNI	−0.128	0.879	0.824–0.938	<0.001
Postoperative ICU stay	0.372	1.451	1.126–1.871	<0.001

Beta coefficient = regression coefficient of multivariate logistic regression; OR, odds ratio; CI, confidence interval. The optimal penalization value (λ) of the LASSO model was 0.032.

### 3.4 Construction and multidimensional validation of the nomogram model

**Figure 2:**
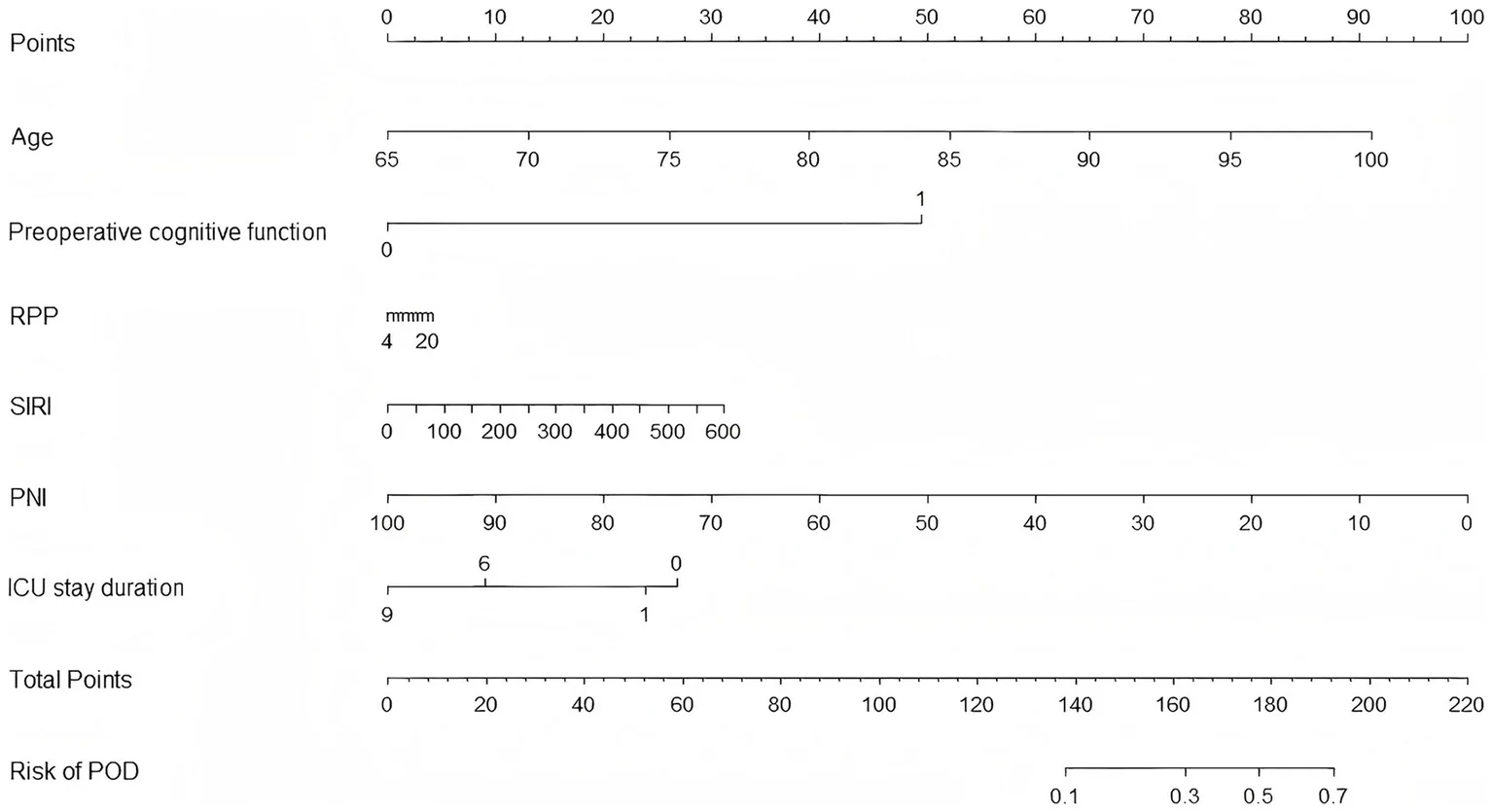
Nomogram for predicting the risk of perioperative delirium in elderly patients with osteoporotic fractures. A nomogram developed from variables screened by LASSO regression and multivariate logistic regression. Included predictors are age, preoperative cognitive function, rate-pressure product (RPP), systemic inflammation response index (SIRI), prognostic nutritional index (PNI) and postoperative ICU stay duration. Individual POD risk probability is estimated by summing the scores of each indicator and matching the total score to the corresponding risk scale.

Based on the results of the multivariable regression, a clinical nomogram model was constructed for the individualised prediction of POD probability. This model transforms complex mathematical formulas into a visual risk-scoring tool. In the nomogram, physicians need only map the patient’s measured indicators to the corresponding score scales, calculate the total score and visually determine the individualised probability of the patient developing postoperative delirium (see Figure 2).

**Figure 3:**
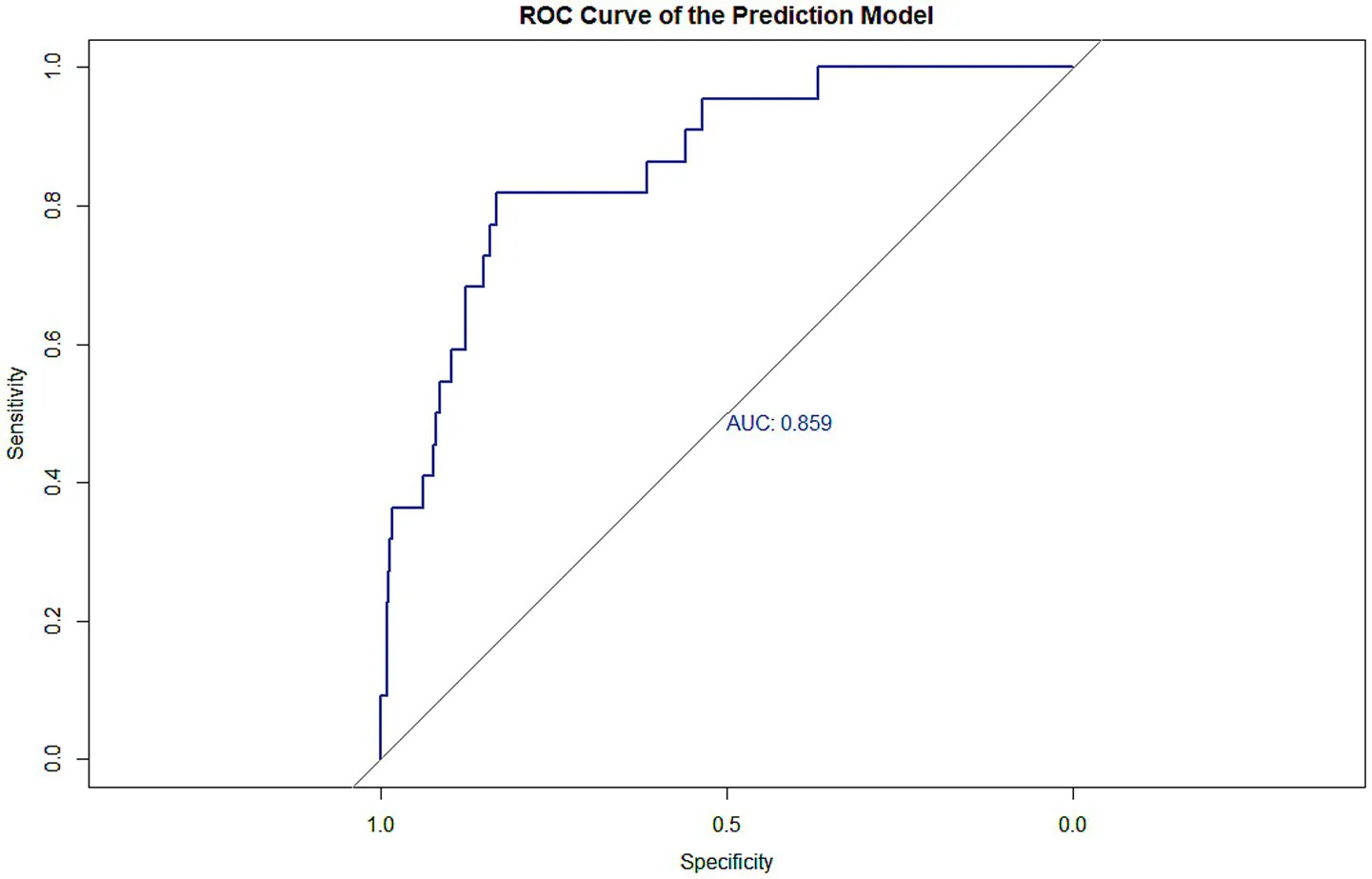
Receiver operating characteristic (ROC) curve of the prediction model. The ROC curve for assessing the discriminative ability of the nomogram. The area under the curve (AUC) was 0.859 (95% CI: 0.782–0.916).

**Figure 4:**
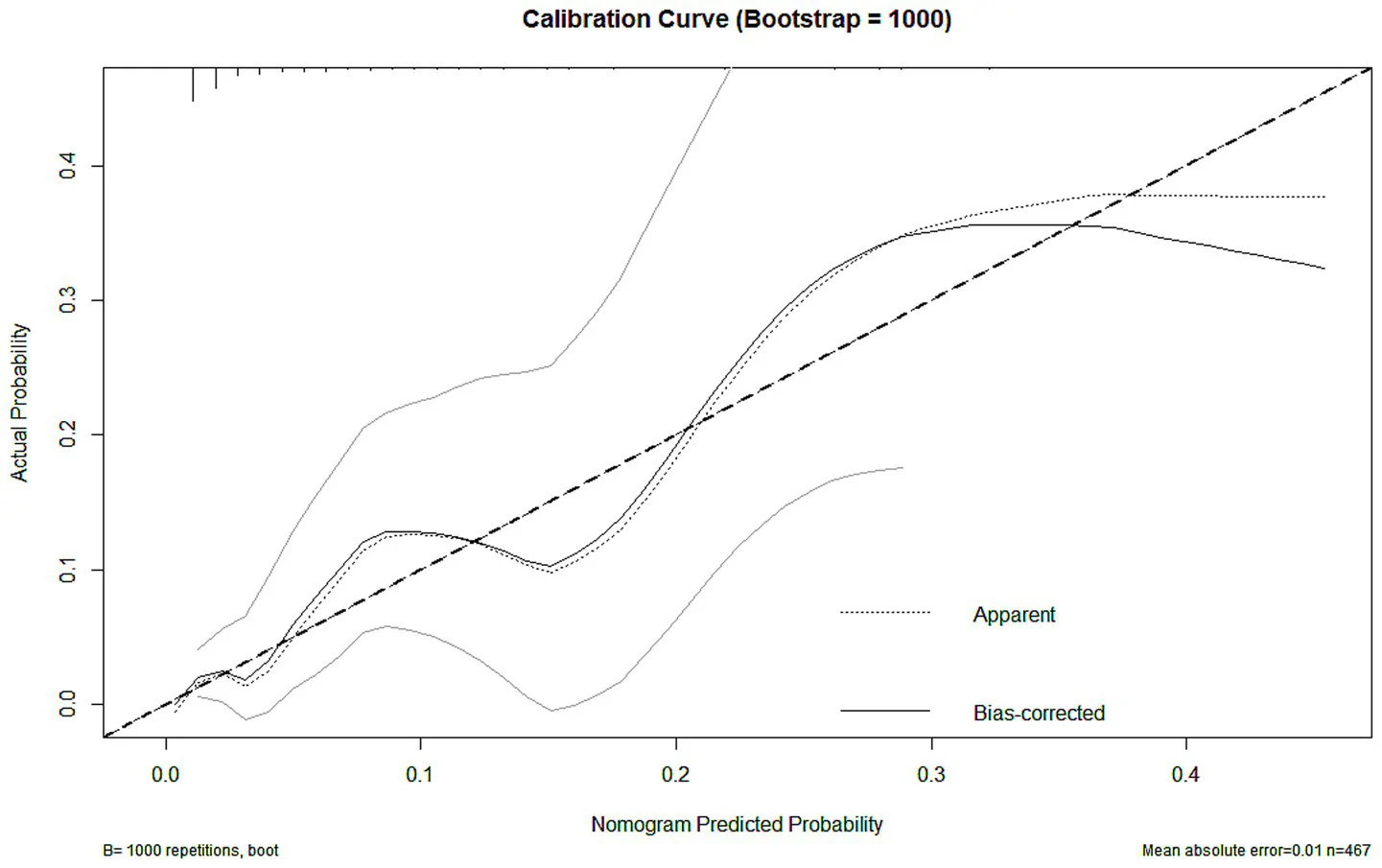
Calibration curve of the nomogram model (Bootstrap = 1,000). Calibration curve for internal validation of the nomogram based on 1,000 Bootstrap resamples. The X-axis shows model-predicted POD probability, and the Y-axis shows the observed POD probability. The diagonal dashed line stands for ideal prediction, and the solid line represents bias-corrected predictive performance. The curve is generally aligned with the ideal diagonal line, showing acceptable consistency between predicted and observed POD probability.

**Figure 5:**
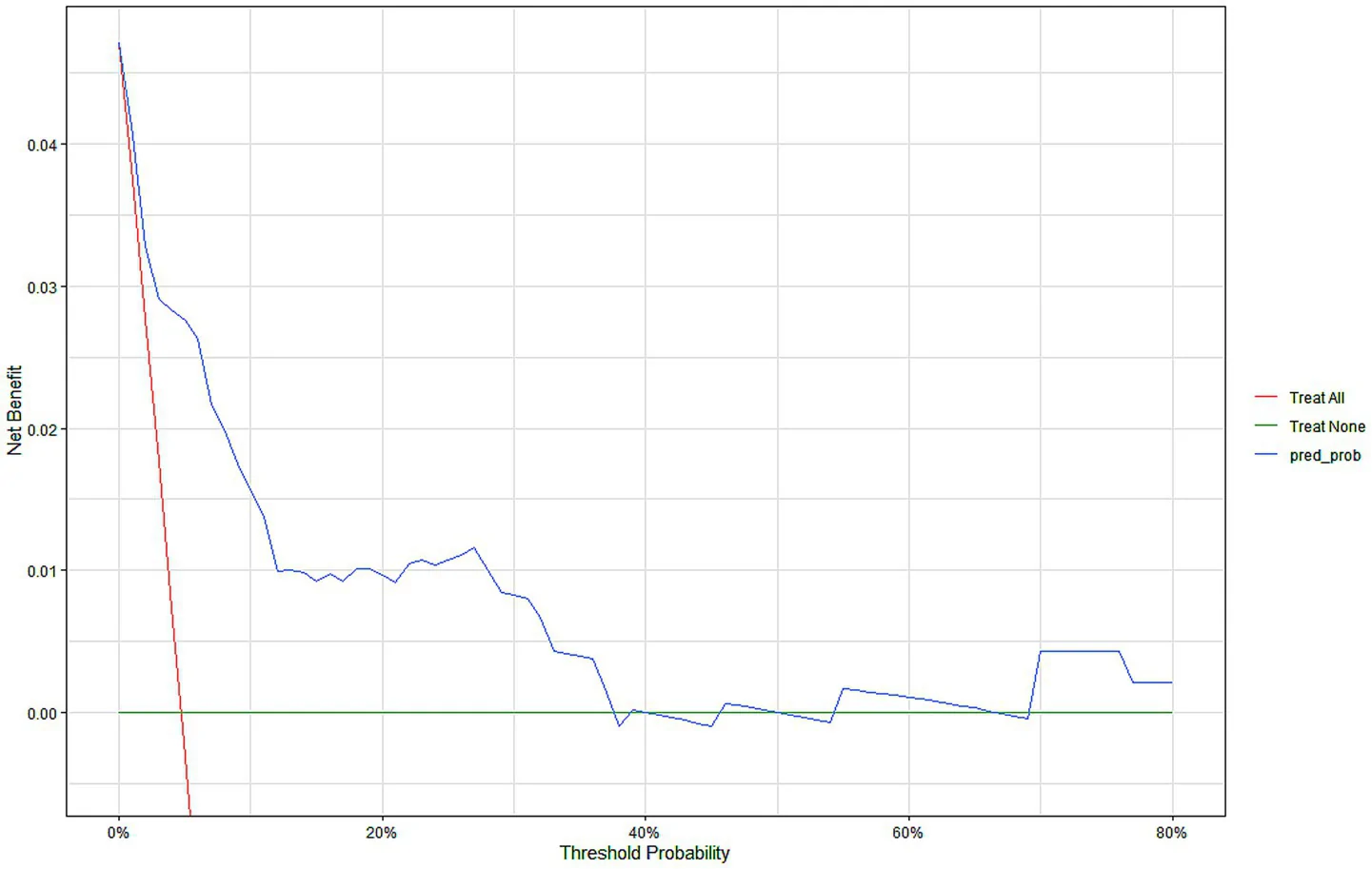
Decision curve analysis (DCA) of the nomogram for predicting the risk of perioperative delirium. Decision curve analysis (DCA) for the nomogram. The Y-axis indicates net clinical benefit and the X-axis indicates threshold probability. The blue line denotes net benefit when using the nomogram for clinical decision-making; the red line represents the “Treat All” strategy and the green line represents the “Treat None” strategy. Across a wide range of threshold probabilities, the curve of the nomogram lies above the two reference curves, showing measurable net clinical benefit.

In the model performance evaluation:

1) Discrimination: Receiver operating characteristic curve analysis showed that the nomogram model presented favourable discriminative ability. The AUC was 0.859 (95% CI: 0.782–0.916) (see Figure 3). At the optimal cut-off value, the sensitivity was 81.82% (95% CI: 60.93–93.39%) and the specificity was 84.42% (95% CI: 77.85–89.34%). The F1 score was 0.724 (95% CI: 0.561–0.837), and the Brier score was 0.093 (95% CI: 0.065–0.128). Compared with relying solely on age or a single risk factor, this multi-parameter model achieved higher sensitivity and specificity.
2) Calibration: The internal calibration curve generated by 1,000 bootstrap resamples showed that the model-predicted probability of POD was highly consistent with the observed incidence, with the scatter points closely distributed around the ideal diagonal line (see Figure 4). The Hosmer–Lemeshow test showed no statistically significant difference (*p* > 0.05).
3) Clinical utility: Decision curve analysis indicated that, over a wide range of threshold probabilities, making clinical intervention decisions based on this nomogram model, compared with the “treat-all” or “treat-none” strategies, resulted in the decision curve remaining above the two baseline curves (see Figure 5), suggesting potential net clinical benefit for clinical decision-making.

To further assess model robustness, several complementary statistical evaluations were performed. Collinearity assessment among inflammatory and nutritional indices revealed variance inflation factors ranging from 1.12 to 2.18, indicating no substantial multicollinearity. Beyond the original 10-fold cross-validation, additional 5-fold and leave-one-out cross-validation yielded AUCs of 0.851 (95% CI: 0.774–0.908) and 0.863 (95% CI: 0.789–0.921), respectively. The Brier score was 0.093 (95% CI: 0.065–0.128), and the integrated calibration index was 0.021, supporting favourable calibration. The precision–recall (PR) curve demonstrated a PR-AUC of 0.812, reflecting reliable performance despite the modest event rate. Sensitivity analysis excluding patients with minor missing covariates (<5%) produced consistent results (AUC change <0.03). Sensitivity analyses adjusting for anaesthesia type, cumulative perioperative sedative dosage, intraoperative BIS anaesthetic depth, postoperative analgesic protocols, opioid dose, intraoperative hypotension and baseline frailty yielded consistent model performance (AUC change <0.02, all *p* > 0.05), suggesting that residual perioperative confounding did not materially alter the predictive utility of the nomogram. Subgroup analyses showed comparable discriminative ability across age strata (<75 vs. ≥ 75 years; AUC 0.847 vs. 0.866) and fracture types (hip vs. non-hip fractures; AUC 0.862 vs. 0.851). Finally, clinical reclassification analysis demonstrated incremental value over age alone, with a net reclassification improvement of 0.241 (95% CI: 0.112–0.369) and an integrated discrimination improvement of 0.094 (95% CI: 0.037–0.151).

## 4 Discussion

This study utilised a machine learning-assisted LASSO regression algorithm to integrate patients’ physiological stress (RPP), systemic inflammatory and immune status (SIRI) and nutritional frailty (PNI) at admission, together with traditional medical history characteristics, to construct and internally validate a multidimensional nomogram model for predicting POD in older patients with osteoporotic fractures. This model demonstrated acceptable predictive performance.

### 4.1 Interpretation of predictive markers: correlation between stress, inflammation, frailty and perioperative delirium

The favourable predictive performance of the established model is attributed to the included indicators, which are closely correlated with three major clinical conditions associated with POD. First, a close correlation is observed between perioperative physiological stress, autonomic nervous system disturbance and POD risk. Acute stress is commonly triggered by severe pain secondary to fracture trauma. Admission RPP, an indicator reflecting myocardial oxygen consumption and sympathetic tone [8], was identified as a strong predictive marker for POD. Excess catecholamine release related to heightened sympathetic activity is correlated with impaired blood–brain barrier endothelial function and reduced cerebral microcirculatory perfusion, which are associated with an increased risk of POD.

Second, a correlation is found between systemic immune-inflammatory responses and POD occurrence, which is consistent with the neuroinflammation hypothesis. One of the included parameters, SIRI, reflects both destructive inflammatory responses mediated by neutrophils and monocytes and immune regulatory dysfunction related to lymphocytes and demonstrates better performance in reflecting systemic immune imbalance than routine white blood cell counts (17, 18). Elevated levels of peripheral pro-inflammatory cytokines are correlated with altered blood–brain barrier permeability, subsequent microglial activation and disrupted synaptic transmission, all of which are associated with a higher probability of POD.

Third, nutritional status and physical reserve are also closely associated with POD risk. Delirium is often regarded as a decompensated state of the brain following multiple adverse insults, and the body’s defensive capacity is largely determined by physical reserve (23). In this model, PNI serves as a valuable predictive marker in this model, indicating that poor nutritional status, such as hypoalbuminaemia, is correlated with weakened endogenous antioxidant defence and altered vascular osmotic pressure (24). Combined with neuronal reserve decline caused by pre-existing cognitive impairment or dementia in older populations, suboptimal physical reserve is associated with increased vulnerability to POD (19, 20).

Fourth, baseline vulnerability and prolonged postoperative ICU stay are also correlated with POD risk, which is in line with the widely recognised predisposing–precipitating framework. Advanced age and preoperative cognitive dysfunction reflect decreased synaptic plasticity and impaired neurocognitive reserve in the central nervous system, representing inherent high-risk characteristics for POD. In addition, prolonged postoperative ICU stay indicates severe post-traumatic physiological disturbance, such as haemodynamic instability. Multiple stressors within the ICU environment, including sleep deprivation, equipment alarms, continuous light stimulation and physical restraint, may induce sensory overload in vulnerable older patients, and such factors are correlated with increased POD risk. Notably, a potential reverse causality exists between postoperative ICU stay and POD. On the one hand, prolonged ICU stay and the corresponding environmental stress are correlated with elevated POD risk. On the other hand, the occurrence of POD and severe systemic conditions in affected patients may also lead to extended ICU admission. This bidirectional relationship may introduce interpretative bias. This model incorporates ICU stay as a marker of cumulative perioperative physiological disturbance rather than as a causal determinant. The temporal alignment – with ICU stay measured before most POD episodes – and the sensitivity analyses support its predictive utility, although caution is warranted when interpreting directionality.

### 4.2 Methodological advantages and clinical application

At the methodological level, traditional studies have mostly used stepwise regression to screen variables. In contrast, this study introduced LASSO penalised regression, which precisely dehydrated high-dimensional data through L1 regularisation and eliminated noise variables with strong multicollinearity, thereby ensuring the model’s generalisability and parsimony in small-to-medium sample sizes (14). Combined with 1,000 bootstrap resamples, the robustness of the model was further strengthened.

The nomogram transforms complex statistical models into an intuitive bedside tool. Physicians need only retrieve routinely accessible indicators such as RPP, SIRI and PNI and combine them with the patient’s medical history to quickly obtain the individualised probability of POD. The performance of the DCA curve suggests that the model may help guide the medical team to implement pre-emptive strategies, such as optimising analgesia, providing preoperative nutritional intervention and shortening ICU stay, thereby helping to prevent delirium and support more precise allocation of medical resources.

### 4.3 Comparison with previous perioperative delirium prediction models

Multiple prediction models for POD in older patients with fracture have been developed in recent years. A model for patients with femoral neck fractures identified glucose, lactate, oxygen partial pressure and neurological scores as the main predictors, with good predictive efficacy (25). Another study involving patients with femoral fractures identified tranexamic acid use, preoperative glucose and lymphocyte count as independent risk factors, with moderate discriminative ability (26). Moreover, a model derived from a meta-analysis summarised traditional risk factors, including age, history of stroke, cognitive impairment and hypoalbuminaemia, and achieved excellent predictive performance during validation (10). Unlike the above models, which primarily relied on single metabolic, haematological or conventional clinical variables, our prediction model incorporated RPP, SIRI and PNI, three composite indicators reflecting haemodynamics, systemic inflammation and nutritional status. These markers are convenient to obtain in routine clinical practise. In addition, preoperative cognitive dysfunction, a well-recognised risk factor in previous studies, was also included in our model. Collectively, our nomogram combines classic risk factors with novel composite biomarkers, thereby enriching the existing POD prediction tools for older patients with osteoporotic fractures.

### 4.4 Limitations and prospects

Several limitations of this study should be acknowledged. First, because this was a retrospective single-centre observational study, potential selection bias is unavoidable. All participants were recruited from a single tertiary hospital, and specific populations may have been excluded according to the inclusion and exclusion criteria, thereby reducing the representativeness of the study sample. Second, the number of patients with POD was relatively small. A low events-per-variable ratio is associated with an increased risk of model overfitting, and the predictive performance may therefore be overestimated in the current dataset. Only internal bootstrap validation was performed, whereas independent external validation using additional patient cohorts was not conducted. Accordingly, the generalisability and extrapolation ability of the developed nomogram require further verification in multicentre prospective studies. Third, postoperative ICU stay duration was included as a predictive factor in the final model. A risk of reverse causality exists between this indicator and POD: prolonged ICU admission may be caused by delirium rather than acting solely as a risk factor for delirium. Such causal ambiguity may interfere with the interpretation of the model results. Fourth, substantial heterogeneity was observed in the anaesthesia and surgical protocols among the enrolled patients. Variations in anaesthetic type, anaesthetic depth, intraoperative medications, surgical procedures and postoperative analgesic regimens may influence the incidence of delirium, and these confounding factors were not fully adjusted for in the present analysis. Fifth, the diagnosis of POD was established using the CAM and 3D-CAM scales. Given the dynamic nature of delirium, hypoactive delirium may have been missed during clinical evaluation, which could lead to bias in the ascertainment of outcome events. In addition, all data were extracted retrospectively from EMRs. Data collection was restricted by incomplete original medical documentation and information loss, creating inherent limitations. Finally, only in-hospital POD was assessed in this study. Long-term follow-up of cognitive function after hospital discharge was not performed; therefore, the long-term cognitive consequences of POD in older patients with osteoporotic fractures could not be evaluated.

## 5 Conclusion

In summary, this study successfully constructed a nomogram model for predicting the risk of POD in older patients with osteoporotic fractures based on feature selection using LASSO regression. These biomarkers were valuable predictive indicators reflecting the combined clinical status of stress, inflammation and physical frailty. Notably, this nomogram has undergone only internal validation. Its generalisability and clinical applicability still require verification in subsequent external cohorts.

## Data Availability

The original contributions presented in the study are included in the article/Supplementary material, further inquiries can be directed to the corresponding author.
