# Supplementary material for: Development and validation of a nomogram prediction model for perioperative delirium in older patients with osteoporotic fractures based on LASSO regression and multiple parameters
Source: Front Med (Lausanne). 2026 Sep 3;13:1869420. doi: 10.3389/fmed.2026.1869420 (PMC13581865; doi:10.3389/fmed.2026.1869420)
Supplement: Supplementary file 1 [file Table_1.docx]

**Supplementary Table S1. Overview of all initially included candidate variables.**

| **Variable name** | **Operational definition** | **Clinical rationale for inclusion** | **Distribution (POD group, N=22) / Non-POD group (N=154)** | **Performance in LASSO selection** |
| --- | --- | --- | --- | --- |
| Age (years) | Chronological age at admission | Advanced age is a well-recognized predisposing factor for POD | 84.27±7.81 / 70.29±3.90 | Retained, non-zero coefficient |
| Sex | Male / Female | Gender may affect stress response and inflammatory status | 4/18 / 46/108 | Excluded, coefficient shrank to zero |
| BMI (kg/m²) | Body mass index calculated by height and weight | Reflects general physical status and nutritional baseline | 24.15±3.22 / 23.86±2.97 | Excluded, coefficient shrank to zero |
| Smoking history | Current / Former / Never smoking | Tobacco use affects cerebral vascular function | 5/17 / 32/122 | Excluded, coefficient shrank to zero |
| Drinking history | Current / Former / Never drinking | Alcohol history correlates with cognitive fluctuation | 3/19 / 27/127 | Excluded, coefficient shrank to zero |
| Hypertension | Presence or absence of preoperative hypertension | Chronic cardiovascular disease increases perioperative risk | 13/9 / 81/73 | Excluded, coefficient shrank to zero |
| Diabetes mellitus | Presence or absence of preoperative diabetes | Metabolic disorder impairs cerebral microcirculation | 6/16 / 42/112 | Excluded, coefficient shrank to zero |
| Cerebrovascular disease | History of stroke or transient ischemic attack | Pre-existing brain injury elevates POD risk | 7/15 / 39/115 | Excluded, coefficient shrank to zero |
| Preoperative cognitive dysfunction/dementia | Diagnosed via MMSE and clinical evaluation | Impaired neural reserve is a core POD predisposing factor | 4/18 / 0/154 | Retained, non-zero coefficient |
| ASA physical status | Class I–IV | Reflects overall preoperative physical tolerance | Class III–IV:15 / 78 | Excluded, coefficient shrank to zero |
| Fracture type | Hip / Vertebral / Other limb | Fracture site correlates with trauma severity | 15/5/2 / 97/42/15 | Excluded, coefficient shrank to zero |
| Interval from fracture to surgery (d) | Days from fracture onset to operation | Prolonged preoperative waiting increases stress | 4.00[3.00,6.00] / 3.00[2.00,4.00] | Excluded, coefficient shrank to zero |
| Anesthesia method | General / Regional / Combined | Anesthesia modality may affect neurological status | 14/5/3 / 84/44/26 | Excluded, coefficient shrank to zero |
| Operation duration (min) | Total surgical time | Longer operation indicates stronger surgical stress | 86.82±61.62 / 92.31±52.62 | Excluded, coefficient shrank to zero |
| Intraoperative blood loss (ml) | Cumulative intraoperative blood loss | Hemodynamic disturbance increases POD risk | 354.55±272.08 / 90.62±104.07 | Excluded, coefficient shrank to zero |
| Postoperative ICU stay (h) | Duration of postoperative ICU admission | Reflects perioperative stress and environmental stimuli | 0.09[0.00,0.00] / 0.00[0.00,0.00] | Retained, non-zero coefficient |
| Admission RPP (mmHg·bpm) | Heart rate × systolic blood pressure | Quantifies perioperative sympathetic stress response | 11207.86±2826.76 / 9885.65±1731.68 | Retained, non-zero coefficient |
| SIRI | (Neutrophil × Monocyte) / Lymphocyte | Marker of systemic immune-inflammatory imbalance | 3.23[2.20,4.89] / 1.15[0.67,2.05] | Retained, non-zero coefficient |
| NLR | Neutrophil / Lymphocyte | Routine inflammatory and immune index | 6.54[4.14,11.62] / 2.70[1.87,4.09] | Excluded, coefficient shrank to zero |
| PNI | Albumin + 5×lymphocyte count | Evaluates nutritional status and physical reserve | 43.40±3.27 / 50.94±6.30 | Retained, non-zero coefficient |
| MMSE score | Mini-Mental State Examination score | Quantitative baseline cognitive function | 62.36±5.72 / 75.87±4.96 | Excluded, coefficient shrank to zero |
| Frailty status | Robust / Pre-frail / Frail | Geriatric frailty reflects overall physiological vulnerability | 4/11/7 / 67/72/15 | Excluded, coefficient shrank to zero |
| ADL score | Activities of Daily Living score | Evaluates functional dependency | 61.24±7.39 / 78.51±6.84 | Excluded, coefficient shrank to zero |
| Intraoperative hypotension | Presence or absence | Hemodynamic instability during surgery | 6/16 / 32/122 | Excluded, coefficient shrank to zero |

Note: Continuous variables are presented as mean ± standard deviation or median [interquartile range]; categorical variables are presented as case number. Variables with non-zero coefficients after LASSO penalization were retained for subsequent multivariate logistic regression analysis.
